# Homoarginine and Mortality in Pre-Dialysis Chronic Kidney Disease (CKD) Patients

**DOI:** 10.1371/journal.pone.0072694

**Published:** 2013-09-04

**Authors:** Pietro Ravani, Renke Maas, Fabio Malberti, Paola Pecchini, Maren Mieth, Robert Quinn, Giovanni Tripepi, Francesca Mallamaci, Carmine Zoccali

**Affiliations:** 1 Department of Medicine and Faculty of Medicine, University of Calgary, Calgary, Alberta, Canada; 2 Institute of Clinical and Experimental Pharmacology and Toxicology, Friedrich-Alexander Universität Erlangen-Nürnberg, Nuremberg, Germany; 3 Divisione di Nefrologia, Azienda Istituti Ospitalieri di Cremona, Cremona, Italy; 4 Divisione di Nefrologia, Ospedali Riuniti and CNR IBIM, Reggio Calabria, Italy; University of São Paulo School of Medicine, Brazil

## Abstract

**Background and Aims:**

Homoarginine, a precursor of nitric oxide, is an inverse predictor of death in dialysis patients and in subjects with cardiovascular disease and normal kidney function but its relationship with clinical outcomes in chronic kidney disease (CKD) patients not yet on dialysis is unknown.

**Design, setting, participants and measurements:**

We enrolled 168 consecutive predialysis CKD patients (Age: 70±11 yrs; 26% Diabetics; eGFR 34±18 ml/min/1.73 m^2^) referred to a tertiary care centre and measured laboratory data on kidney function and cardiovascular risk factors. We modeled progression to dialysis or death as a function of homoarginine, using Cox’s regression, accounting for clinical characteristics, baseline levels of kidney function, and markers of inflammation.

**Results:**

On crude and adjusted analyses homoarginine was directly associated with the eGFR and patients with more compromised renal function exhibited lower homoarginine levels. Furthermore homoarginine was also independently related to L-arginine, serum albumin and body mass index, and inversely related to proteinuria, C-reactive protein and age. During the study (follow up median time 4 years, inter-quartile range 1.7 to 7.0 years) 56 patients started dialysis and 103 died and homoarginine was a strong inverse predictor of the incidence rate of both outcomes (P = 0.002 and P = 0.017).

**Conclusions:**

Homoarginine declines with advancing renal disease and is inversely related to progression to dialysis and mortality. The nature of the link between homoarginine and clinical outcomes is amenable to testing in clinical trials.

## Introduction

Reduced nitric oxide (NO) bioavailability is a major risk factor for cardiovascular disease and progression to kidney failure in patients with chronic kidney disease (CKD) [1]–[3]. High levels of structural analogs of the main precursor of NO L-Arginine, are considered as a main mechanism impairing NO synthesis in this condition[3]; [4]. Accumulation of endogenous inhibitors of NO synthase apart, low homoarginine levels associated with decreased renal function [5] may contribute to reduce NO synthesis in CKD. This lysine-derived cationic amino acid may increase NO bioavailability by multiple mechanisms [6]. Involvement of homoarginine in the regulation of vascular function in man is supported by the direct association between the plasma levels of this aminoacid with the hemodynamic response to ischemia in the forearm [6]. Even though observations showing compromised tubular homoarginine reabsorption in the remnant kidney model were made in the mid-nineties, there has been only sparse interest on this aminoacid in clinical research in CKD. Recently März et al. [7], noted a strong, direct association between levels of homoarginine and estimated glomerular filtration rate (eGFR) in a large cohort of patients with substantially normal renal function (average eGFR = 81 ml.min^−1^.1.73 m^2^) referred for coronary angiography. This finding indicates that in the normal eGFR range a gradual decrease in circulating homoarginine may occur at progressively lower eGFR levels [7]. Remarkably, the same authors also documented an inverse association between plasma homoarginine, left ventricular systolic dysfunction, and all-cause and cardiovascular mortality in the same cohort, and confirmed this association in a second cohort of hemodialysis patients with type-2 diabetes [8]. Collectively, these findings implicate progressive homoarginine deficiency brought about by declining renal function in the high cardiovascular risk engendered by progressive CKD. Furthermore, low homoarginine may either engender and/or aggravate renal disease via endothelial dysfunction [9], i.e. by a mechanism considered as of primary importance in this condition [9]. However, no data are currently available on the association between eGFR and homoarginine in patients with established CKD or on the link between homoarginine and clinical outcomes in this patient population.

In this study we investigated the relationship between circulating homoarginine with traditional and non-traditional cardiovascular risk factors in an incident cohort of CKD patients and tested the relationship between this aminoacid and all-cause and cardiovascular death, and renal outcomes.

## Materials and Methods

### Protocol

This cohort study was designed to investigate the impact of non-traditional cardiovascular risk factors on renal function decline and patient outcomes in CKD. The study protocol was submitted to the Ethical Committee of our institution, resulted to be in conformity with the ethical guidelines and was specifically approved by the Ethical Committee of Azienda Ospedaliera “Istituti Ospitalieri” di Cremona (Italy). Written informed consent was obtained from each participant.

### Patients

All consecutive subjects 18 year-old or older, with stage 1 to 5 CKD, referred to the outpatient clinic of the Renal Unit of Cremona Hospital from January 2002 until April 2003 were enrolled in the study. Data on baseline characteristics, renal disease causes, previous or actual smoking habit, documented diagnosis of diabetes, cerebro-vascular disease, peripheral artery disease, coronary artery disease, heart failure, neoplasm, and history of hypertension were collected at the first referral and defined based on the diagnosis-related group classification and International Classification of Diseases, Ninth Revision. Patients were subsequently followed regularly with frequency dependent on the levels of kidney function within a structured multi-disciplinary CKD clinic [10]. All patients had to be in clinically stable conditions, and were not expected to start dialysis within six months of study enrolment.

### Laboratory Measurements

All patients were carefully instructed for 24 hours urine collection and additional urine spot for proteinuria assessment. Blood sampling was performed after 20 to 30 min of quiet resting in a semi-recumbent position. A fasting blood sample for serum cholesterol, creatinine, albumin, fibrinogen, C-reactive protein, total homocysteine, calcium, phosphate, total PTH, 25-hydroxy Vit. D, 1,25-hydroxy Vit. D and hemoglobin was obtained from all patients at baseline. Plasma was stored at −80°C until analysis. Glomerular filtration rate (GFR) was estimated using the MDRD formula (eGFR). Plasma total homocysteine (Abbott Laboratories, Berkshire, England) and serum C-reactive protein (Behring, Scoppito, L’Aquila, Italy) were measured by using commercially available kits.

Plasma concentrations of asymmetric L-dimetylarginine (ADMA), L-homoarginine and L-arginine were determined by liquid chromatography-tandem mass spectrometry (LC/MS/MS) using minor modifications of a method that we previously validated for asymmetric L-dimethylarginine and L-arginine [11], in addition we validated a specific extension of the method for L-homoarginine [12]. To reduce matrix effects like ion suppression and to improve separation of homoarginine from related analytes, an additional chromatographic separation was performed.

### Follow-up and Outcomes

After the initial assessments, laboratory and clinical data including cardiovascular events and death, and the need for dialysis due to end-stage renal disease were accurately recorded. To avoid loss to follow-up, patients were contacted by phone in case they missed any appointment and at the study end date (August 31, 2010) if they were not known to be already on dialysis or dead. Each death was reviewed and assigned an underlying cause by 3 physicians, on the basis of all available medical information. In cases of out-of hospital deaths, family members were interviewed by telephone to ascertain the circumstances surrounding the death [10].

### Statistical Analysis

#### Descriptives

Data are expressed as mean ± standard deviation (SD), median and range or as percent frequency, as appropriate. Variables deviating from the normal distribution were log-transformed in regression analyses.

#### General linear model of homoarginine and eGFR

Plasma homoarginine was modelled as a function of traditional and non-traditional risk factors including clinical data and laboratory measures described above (excluding eGFR) on the basis of their univariate association or clinical plausibility. Since homoarginine was log-transformed for this analysis, the exponential of the corresponding regression coefficients [calculated by elevating the Neper number (2.7183) to the coefficients] are interpreted as percent change in the dependent variable per unit change in the predictor. The independent relationship of homoarginine on eGFR was subsequently tested using linear regression with GFR as response variable.

#### Survival functions

The Cox’s procedure was used to model time to dialysis start and death as a function of homoarginine levels. We fitted model for correlated failure events of different types (dialysis or death), because more than one event could occur for the same subject [13]. In this model which accounts for the competing risk of death, each subject is at risk for two events and observations continue for each subject beyond the first event that occurs [13]. The lack of independence of the failure times was accounted for by using robust variance [14]. The model was stratified by failure type (dialysis and death), allowing each stratum to have its own baseline hazard function and regression coefficients. The largest possible meaningful model considered included clinically consistent variables and interpretable interaction terms identified based on biological plausibility or strength of their univariate relation to the outcome, following the rule of “ten” (one parameter per 10 events) and considering the overall model fit and hazard proportionality. A manual elimination approach was followed, monitoring variations of the exposure regression coefficient to identify variables eligible to be dropped as non-confounders. We determined the functional form of the exposure-outcome relationship by testing linear and non-linear associations of homoarginine with the main study outcomes (progression to dialysis or death) [15]. We obtained predicted values for incidence rates of ESRD and mortality across homoarginine values from the multiple events Cox model at the mean values of the regression covariates. For all models goodness-of-fit and assumptions were assessed using residual analyses and graphically. All analyses were performed using STATA 12 MP (StataCorp, College Station, TX).

## Results

### Patient Characteristics

Table 1 summarizes the demographic and baseline characteristics of the study cohort. The study cohort included 168 subjects, all of Caucasian descent, who were on average 70 year old (±11), prevalently male (62%) and with background cardiovascular disease (57%). Baseline eGFR was 34±18 ml/min/1.73 m^2^. The prevalence of diabetes was 26%.

**Table 1:** Baseline characteristics.

	N = 168
Age (years)	70 (11)
Male sex	104 (62)
Diabetes	43 (26)
Previous CVD	95 (57)
Peripheral vascular disease**	48 (29)
Hypertension (>5 years)	136 (81)
Cancer	20 (12)
Smoking	53 (32)
Body Mass Index (Kg/m^2^)	26.4 (5)
Total cholesterol (mg/dL)	205 (54)
HDL cholesterol (mg/dL)	52 (13)
Triglycerides (mg/dL)	156 (82)
eGFR (ml/min/m^2^)	34 (18)
Proteinuria (g/day)*	0.4 (0.0, 16.1)
25-hydroxy Vit. D (ng/mL)	21(11)
1,25 - hydroxy Vit. D (ng/mL)	20(12)
Total PTH (pg/mL)	117 (114)
Calcium (mg/dL)	9.4 (0.6)
Phosphate (mg/dL)	3.5 (0.9)
Hemoglobin (g/dL)	12.8 (1.6)
Serum phosphate (mg/dL)	3.5 (0.9)
Serum Calcium (mg/dL)	9.3 (0.6)
Serum albumin (g/dL)	3.85 (0.55)
C-reactive protein (mg/L)*	4.0 (0.3, 46.3)
Fibrinogen (mg/dL)	420 (122)
Homocysteine (µmol/L)	26 (17)
L-arginine (µmol/L)*	115 (38, 216)
ADMA (µmol/L)	0.59 (0.10)

Legend. Baseline characteristics of the study population. Mean (SD) and absolute frequencies (%) are reported for quantitative and qualitative variables respectively. For non-normally distributed variables median (range) values are reported (*). HARG: Homo-Arginine; CVD: cardiovascular disease; eGFR: estimated glomerular filtration rate; ADMA: Asymmetric dimetyhlarginine. **Peripheral vascular disease was defined on clinical basis as previous amputation, claudicatio or as documented by angiographic studies.

### Correlates of Homo-Arginine

**Figure 1:**
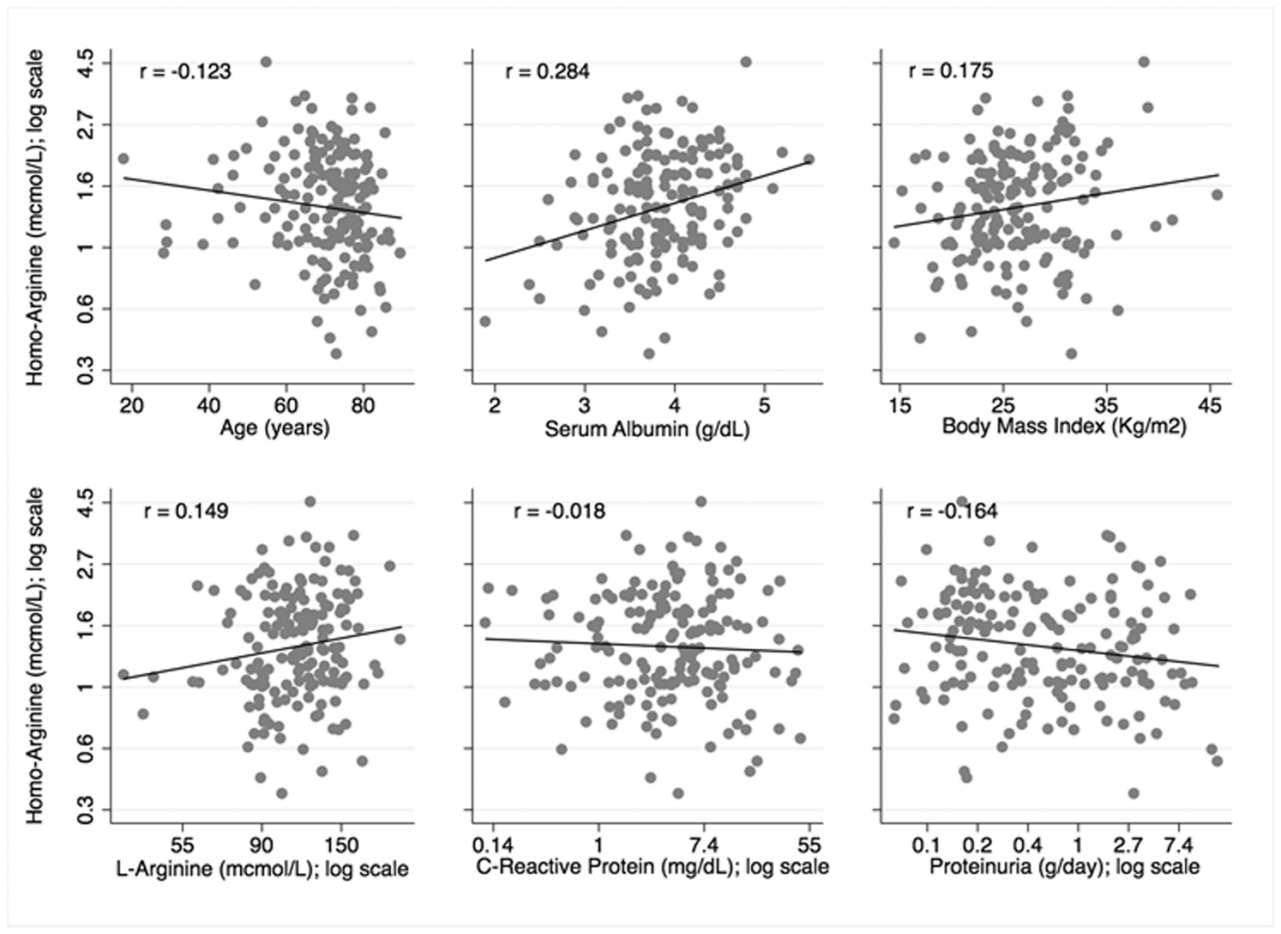
Correlation between homoarginine, age, serum albumin, body mass index, L-Arginine, C-reactive protein and proteinuria.

On crude (**Figure 1**) and adjusted (Table 2) analyses homoarginine was directly related to L-arginine, serum albumin and body mass index, and inversely related to proteinuria, C-reactive protein and age. There was no association between asymmetric dimetyhlarginine and homoarginine. Homoarginine values were 44% greater in men than in women and 18% greater in diabetic patients than in non-diabetic patients, but 14% lower in those with peripheral vascular disease. Forcing smoking into the model did not change the results of multivariate modelling (data not shown).

**Table 2:** Linear Model of Plasma Homoarginine (µmol/L)*.

	Coefficient	95% CI	Beta^†^	P value
L-arginine (µmol/L)*	0.355	0.140, 0.569	0.219	0.001
Proteinuria (g/day)*	−0.078	−0.121, −0.035	−0.282	<0.001
Serum Albumin(g/dL)	0.121	0.003, 0.241	0.154	0.045
C-Reactive Protein(mg/dL)*	−0.072	−0.132, −0.012	−0.178	0.018
Homocysteine(µmol/L)	0.003	−0.001, 0.006	0.121	0.074
Body Mass Index(Kg/m^2^)	0.028	0.016, 0.040	0.327	<0.001
Age (years)	−0.005	−0.011, −0.001	−0.152	0.039
Male (vs. female) sex	0.367	0.243, 0.491		<0.001
Presence of diabetes	0.165	0.026, 0.303		0.020
Peripheral vasculardisease	−0.148	−0.279, −0.017		0.027

Legend: (*) indicates variables that were log-transformed; the regression coefficients estimate the average change in the log values of the response (homo-arginine) per unit change in the predictor (log-unit change [2.72 units on the natural scale µmol/L] if log-transformed); 95% CI indicates 95% Confidence Intervals; Beta† indicates the standardized regression coefficient (not meaningful for the binary variables sex, diabetes and vascular disease). Model R^2^∶0.366; all variance inflating factors <1.1.

### Relationship between eGFR and Homoarginine

**Figure 2:**
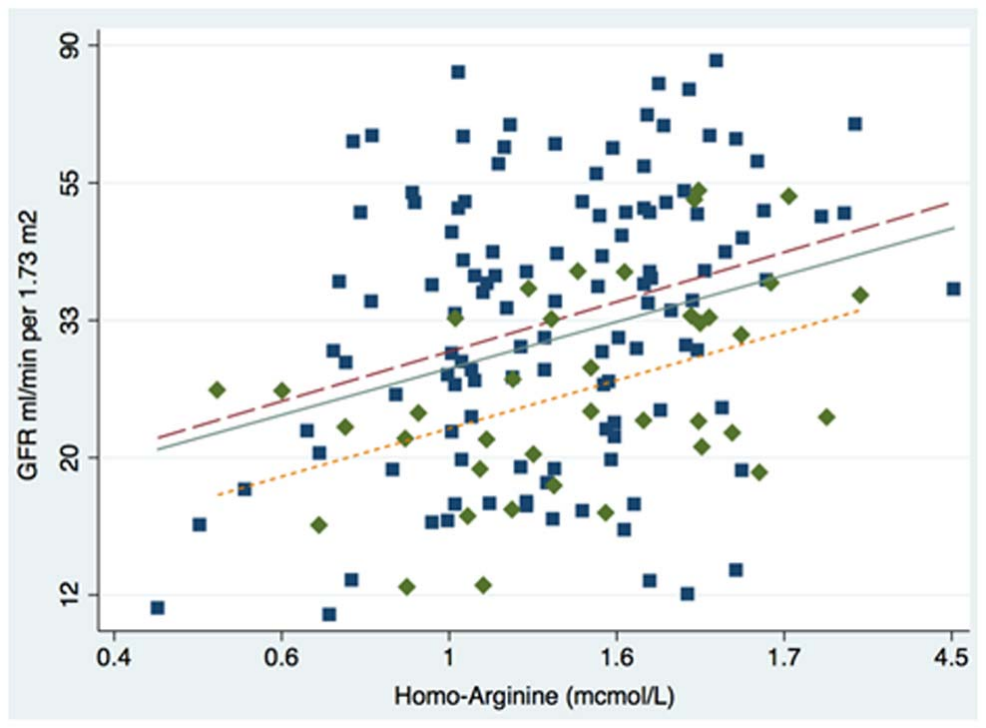
Observed relationship between Glomerular Filtration Rate at baseline (first patient referral to the nephrologist) and homoarginine (log scale), by presence (short dash; squares) and absence of diabetes (long-dash; circles). The solid line represents the linear fit.

There was a log-linear relationship between eGFR and homoarginine values, which was unaffected by gender, but shifted toward lower average values of GFR in diabetic patients. **Figure 2** and Table 3 respectively represent the crude and adjusted relationship between eGFR and homoarginine.

**Table 3:** Linear Model of Glomerular Filtration Rate (ml/min per 1.73 m^2^)* as a function of Homoarginine.

	Coefficient	95% CI	Beta^†^	P value
Homoarginine(µmol/L)*	0.404	0.231, 0.577	0.313	<0.001
Homocysteine(µmol/L)	−0.009	−0.014, −0.005	−0.294	<0.001
ADMA (µmol/L)*	−0.595	−1.072, −0.117	−0.169	0.015
L-arginine (µmol/L)*	−0.388	−0.672, −0.104	−0.230	0.008
Presence of diabetes	−0.296	−0.466, −0.126		0.001

Legend: (*) indicates variables that were log-transformed; the regression coefficients estimate the average change in the log values of the response (Glomerular Filtration Rate [GFR] in ml/min per 1.73 m^2^) per unit change in the predictor (log-unit change [2.72 units on the natural scale µmol/L] if log-transformed); 95% CI indicates 95% Confidence Intervals; Beta^?^ indicates the standardized regression coefficient (not meaningful for the binary variable diabetes); ADMA: asymmetric dimetyhlarginine; Model R^2^∶0.289; all variance inflating factors <1.1.

### Follow-up Data

**Figure 3:**
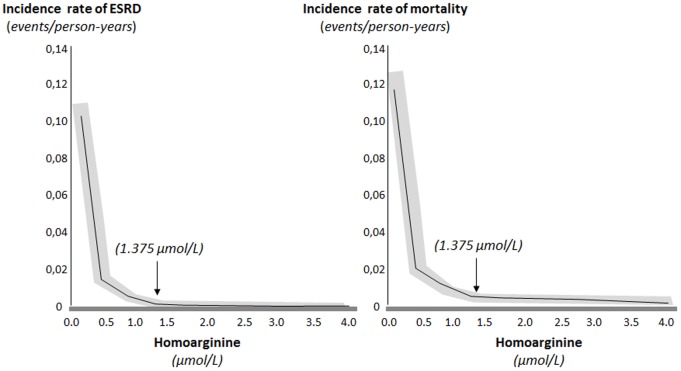
Relationship between homoarginine values (in umol/L) above and below the cut-off of 1.375 umol/L (median value) and predicted adjusted incidence rate of study outcomes at the covariate means (final Cox model summarized in Table 4, Third column). The grey area identifies the 95% confidence interval for the predicted values.

Patients were followed up for a median time of 4 years [inter-quartile range 1.7 to 7.0 years]; 700 patient-years). During the study, 56 patients started dialysis and 103 died (31 after dialysis initiation). Mortality rate was on average 0.15 events per person-years (95% CI from 0.12 to 0.18), significantly higher in patients referred with eGFR <30 ml/min/1.73 m^2^ (0.20 per person-years [95% CI 0.08 to 0.14]) than in patients referred with eGFR ≥30 ml/min/1.73 m^2^ (0.11 per person-years [0.15 to 0.26]; difference 0.09, 95% CI from 0.03 to 0.15 per year). The cause of death was undetermined in 20% of the patients and related to cardiovascular disease in 70% of the patients. There was an inverse linear relationship between homoarginine levels and the risk of progression to dialysis and mortality (the lower homoarginine the higher the risk for adverse outcomes), which was stronger for values below the median (**Fig. 3** **and** **Table 4**). Results for cardiovascular mortality were similar, but the association of homoarginine with this outcome failed to achieve formal statistical significance (P = 0.12).

**Table 4:** Survival data.

Model	Unadjusted HR (95% CI)	Adjusted HR (95% CI)‡	Fully adjusted HR (95% CI)
*ESRD, per 1 µmol/L increase*			
Homoarginine (values <1.375)	0.595 (0.171, 2.062)	**0.161 (0.051, 0.509)**	**0.187(0.061,0.581)**
Homoarginine (values ≥1.375)	0.645 (0.319, 1.308)	**0.533 (0.279, 1.000)**	**0.578(0.319,0.987)**
*Death, per 1 µmol/L increase*			
Homoarginine (values <1.375)	0.468 (0.199, 1.097)	**0.366 (0.160, 0.837)**	**0.366(0.165,0.812)**
Homoarginine (values ≥1.375)	**0.591 (0.357, 0.978)**	**0.582 (0.356, 0.951)**	**0.615(0.386,0.978)**

Legend: Unadjusted and adjusted hazard ratios (HR) and 95% Confidence Intervals (CI) for end-stage renal disease (ESRD) and death associated with 1 unit (*µmol/L*) change in homoarginine. (‡) Relative risks for ESRD adjusted for levels of kidney function and proteinuria; relative risks for death adjusted for levels of kidney function, proteinuria, age, smoking habit, previous cardiovascular disease and diabetes. All models were independent of biomarkers of inflammation (C-reactive protein, fibrinogen, serum albumin), homo-cysteine, hypertension, body mass index and serum lipids, ADMA and L-arginine). Statistically significant estimates are reported in bold.

In fully adjusted models (last column in Table 4), we also forced PTH, phosphate, calcium, 25-hydroxy Vit. D and 1,25-hydroxy Vit. D as potential confounders.

## Discussion

In pre-dialysis CKD patients, plasma homoarginine concentration is associated with the severity of renal dysfunction as well as with biomarkers of protein energy wasting and inflammation (body mass index, serum albumin and C-reactive protein). Furthermore, homoarginine predicts the risk of progression to dialysis and death in CKD.

The kidney is the major site of homoarginine synthesis and disposal, and genetic control of the key enzyme for the biosynthesis of this aminoacid, alanine-glycine aminotransferase. Therefore studying the link between homoarginine and clinical outcomes in CKD is a relevant undertaking to explore the potential implication of this aminoacid in human disease. Extending observations by März in patients with heart failure and substantially normal eGFR [7] and by Drechsler C et al. in patients with CKD [16], our findings in CKD patients provide evidence that homoarginine levels are inversely related with the eGFR across a wide eGFR spectrum spanning from mild to severe CKD and for the first time show that such a link is fully independent of a series of relevant risk factors including asymmetric dimethyl-arginine, a fundamental player in the pathogenetic pathway linking alterations of nitric oxide synthesis and renal dysfunction in humans. In our cohort plasma homoarginine levels were lower than in the healthy, reference population [17] or patients with coronary heart disease and preserved kidney function [8], but distinctly greater than recently reported in hemodialysis patients [7]. This finding further highlights the relevance of kidney function in determining circulating homoarginine. Herein we show for the first time that low homoarginine levels go along with proteinuria, a major renal and cardiovascular risk factor. Homoarginine levels resulted to be sex-dependent and directly related with established biomarkers of protein energy wasting such as body mass index, serum albumin and L-arginine, a key substrate in the metabolic pathway conducive to NO synthesis, although unrelated to the main endogenous inhibitor of NO synthase asymmetric dimethylarginine. These latter associations confirm specifically in pre-dialysis patients findings in hemodialysis patients suggesting that low homoarginine is a marker of negative protein balance and poor nutritional status. Finally, reduced levels of homoarginine in CKD patients with peripheral vascular disease is consistent with the hypothesis that this alteration denotes endothelial dysfunction and signals a high risk for atherosclerosis.

CKD is a typical age-related chronic disease, and our incident CKD cohort had an average age of 71 years. We found that homoarginine is inversely associated with all cause death independent of classical risk factors, risk factors peculiar to CKD and of key players in NO metabolism in this population, namely L-Arginine and of the main endogenous inhibitor of NO synthase, asymmetric dimethylarginine. Homoarginine is a less important substrate for NO synthase than L-arginine. Although consistent with findings from studies in cardiac patients with normal kidney function [8] or diabetic patients on dialysis [9], it is unclear why the plasma levels of the first but not those of the second aminoacid predicted adverse clinical outcomes in the present CKD cohort. Homoarginine levels were particularly reduced in patients with protein energy wasting and inflammation. Although the malnutrition-inflammation complex is considered a major player in the high risk of death and cardiovascular complications of kidney failure, this factor is unlikely to fully account for the predictive power for mortality by low homoarginine. Indeed this link was modestly affected by adjustment for body mass index, serum albumin and C-reactive protein levels. Pregnancy, a physiological condition characterized by a high NO bio-availability and marked peripheral vasodilation, is characterized by a doubling in the plasma homoarginine levels implicating this compound in endothelium mediated, NO-dependent vasodilatation. Although interference with arginine transport mechanisms and/or inhibition with arginase may play a role in the vasodilatation triggered by homoarginine, mechanisms underlying this effect still remain unclear.

Our study has limitations. First, our cohort was relatively small and composed by individuals of Caucasian descent and therefore our observations need to be replicated in other cohorts and in other ethnicities. Second, the observational design of our study does not allow us to draw conclusions about the nature (causal/non casual) of our findings which need to be confirmed in in mechanistic and interventional studies.

In summary, we found that levels of homoarginine are lower in people with reduced kidney function not yet on dialysis and directly related to glomerular filtration rate. Greater levels of homoarginine independently predict longer survival in chronic kidney disease and a reduced risk of progression to dialysis. These observations form the basis for designing clinical trials based on homoarginine supplementation in patients with CKD.
